# Molecular Epidemiology of *Mycobacterium tuberculosis* in Mexico

**DOI:** 10.3390/microorganisms13112453

**Published:** 2025-10-25

**Authors:** Luis M. Rodríguez-Martínez, Jose L. Chavelas-Reyes, Carlo F. Medina-Ramírez, Eli Fuentes-Chávez, Zurisaday S. Muñoz-Troncoso, Ángeles G. Estrada-Vega, Enrique Rodríguez-Díaz, Diego Torres-Morales, María G. Moreno-Treviño, Josefina G. Rodríguez-González

**Affiliations:** 1 Escuela de Medicina, Universidad de Monterrey, Monterrey 64460, Mexico; zurisaday.munoz@udem.edu (Z.S.M.-T.); angeles.estrada@udem.edu (Á.G.E.-V.); enrique.rodriguezd@udem.edu (E.R.-D.); diego.torresm@udem.edu (D.T.-M.); maria.moreno@udem.edu (M.G.M.-T.); 2 Centro de Estudios e Investigaciones Interdisciplinarios, Universidad Autónoma de Coahuila, Saltillo 25280, Mexico; eliffuentes12@gmail.com (E.F.-C.); josefina_rodriguez@uadec.edu.mx (J.G.R.-G.); 3 Centro de Biotecnología Genómica, Instituto Politécnico Nacional, Reynosa 88710, Mexico; jl.chavelas10@gmail.com (J.L.C.-R.); francomedina000@gmail.com (C.F.M.-R.)

**Keywords:** tuberculosis, molecular epidemiology, drug resistance, *Mycobacterium tuberculosis* lineages, genetic diversity

## Abstract

Tuberculosis (TB), caused by *Mycobacterium tuberculosis*, continues to be a leading cause of morbidity and mortality in Mexico, with more than 20,000 new cases annually and a rising proportion of drug-resistant strains. This work addresses the molecular epidemiology of TB in the Mexican context, emphasizing its role in understanding transmission, genetic diversity, and resistance mechanisms. To achieve this, we reviewed molecular typing approaches including spoligotyping, Mycobacterial Interspersed Repetitive Unit–Variable Number Tandem Repeat (MIRU-VNTR) analysis, and whole-genome sequencing (WGS), which have been applied to characterize circulating lineages and identify drug-resistance-associated mutations. The results indicate that the Euro-American lineage (L4) predominates across the country, although significant regional variation exists, with Haarlem, LAM, T, and X sub lineages dominating in different states, and occasional detection of Asian (L2) and Indo-Oceanic (L1) lineages. Key resistance mutations were identified in *katG*, *rpoB*, *pncA*, and *gyrA*, contributing to the emergence of multidrug-resistant (MDR) and extensively drug-resistant (XDR) strains, particularly in border and marginalized regions. These findings highlight how social factors, such as migration, urban overcrowding, and comorbidities including diabetes and HIV, influence transmission dynamics. We conclude that integrating molecular tools with epidemiological surveillance is crucial for strengthening public health strategies and guiding interventions tailored to Mexico’s heterogeneous TB burden.

## 1. Introduction

Tuberculosis (TB) is an infectious disease caused by *Mycobacterium tuberculosis (M. tuberculosis)*. It remains one of the leading causes of morbidity and mortality worldwide, with approximately 10 million new cases and 1.4 to 1.5 million deaths reported annually [1].

*M. tuberculosis* is an acid-fast, obligatory aerobic, slow-growing bacillus characterized by a cell wall rich in mycolic acids that provides high resistance and persistence capacity. Its genome, although highly conserved, exhibits variations that define lineages with different levels of virulence and transmissibility [2]. Among its main virulence factors are the ESAT-6/CFP-10 secretion system and trehalose dimycolate, which enable the bacterium to evade the immune response and survive within macrophages. Infection begins through the inhalation of bacilli, which can remain latent within granulomas or cause active disease upon reactivation [3].

In Mexico, TB also represents a significant public health concern, with approximately 20,000 cases reported annually and an incidence that varies according to geographic and socioeconomic regions [4]. To better understand the dynamics of this infection, specialized disciplines such as molecular epidemiology are employed. This field has emerged as an essential tool to elucidate transmission patterns, genetic diversity, and the emergence of drug-resistant strains [5].

Molecular epidemiology integrates techniques such as spoligotyping, Mycobacterial Interspersed Repetitive Unit–Variable Number Tandem Repeat (MIRU-VNTR) analysis, and whole-genome sequencing (WGS) to identify predominant genetic families, which may be associated with distinct patterns of virulence and resistance [6,7]. This information is crucial for designing control strategies tailored to the Mexican context, where factors such as migration and rapid urbanization contribute to the spread of the disease [8].

In Mexico, the geographic distribution of *M. tuberculosis* lineages is heterogeneous, with certain sub-lineages showing regional predominance. Several studies have documented a higher frequency of drug-resistant strains in border regions, which may be linked to cross-border migration flows and greater exposure to areas with a high endemic burden of multidrug-resistant TB [9]. In contrast, other circulating lineages are associated with sustained community transmission, a pattern commonly observed in marginalized urban areas [10]. Molecular epidemiology has revealed that these patterns not only reflect the evolutionary history of the strains but also mirror social dynamics such as overcrowding and limited access to healthcare services [11]. These findings underscore the need for region-specific public health policies for TB control, including the implementation of rapid molecular diagnostics with mutation panels adapted to the national context, therapeutic adherence support programs in indigenous and rural communities, and the strengthening of genomic surveillance to anticipate the spread of resistant strains [12].

A critical challenge in Mexico is the increasing resistance to antituberculosis drugs, particularly multidrug-resistant TB (MDR-TB) and extensively drug-resistant TB (XDR-TB). Molecular studies have identified mutations in genes such as *rpoB* and *katG*, as well as the circulation of strains with complex resistance profiles (Table 1). These genetic alterations, which occur primarily in antibiotic target genes, explain the loss of efficacy of standard treatments and form the basis of the resistance patterns observed across different regions of the country [13,14,15].

The spread of these strains has been associated with incomplete treatments, delayed diagnosis, and nosocomial transmission [16]. Genotyping has shown that certain MDR strains form epidemiological clusters, suggesting active transmission rather than individually acquired resistance [17]. Beyond bacterial diversity, the interaction between *M. tuberculosis* and the human host plays a key role in TB epidemiology. In Mexico, research has explored genetic polymorphisms in indigenous and mestizo populations that may influence susceptibility to infection or progression to active disease [18,19,20].

## 2. Systematic Review and PRISMA Framework

This systematic review was designed and reported in accordance with the PRISMA 2020 statement. We performed comprehensive research in PubMed, Scopus, Google Scholar, and the MDPI Open Access Database covering the period from January 2010 to April 2025. The primary Boolean string was as follows: (*Mycobacterium tuberculosis* OR MTB) AND (Mexico OR Mexican) AND (spoligotyping OR MIRU-VNTR OR “whole genome sequencing” OR WGS OR lineage OR genotype OR sub lineage OR drug resistance). No language filters were imposed at the search stage; however, only peer-reviewed, indexed publications were considered eligible. Reference lists of included papers were hand-screened to identify additional records.

The initial search retrieved 2318 records worldwide. After automated and manual deduplication, 1420 unique records remained for title/abstract screening. Of these, 68 full-text articles referring to *M. tuberculosis* clinical isolates from Mexico were assessed for eligibility. Nineteen studies met all inclusion criteria and were incorporated into the final synthesis (*k* = 19). Inclusion criteria required: (i) confirmed clinical isolates obtained in Mexico; (ii) molecular typing by spoligotyping, MIRU-VNTR, or WGS; and (iii) quantitative reporting of lineage distribution and/or drug-resistance metrics with explicit denominators. We excluded datasets with <10 isolates, reviews/commentaries without primary data, and studies composed exclusively of MDR cases from the pooled prevalence calculations (though such data could be used descriptively for mutation summaries when applicable). To avoid double-counting, multi-region aggregates reported as “National total” were not used for pooled numerators/denominators when state-level data were available.

Data extraction captured study year(s), state/region, sampling frame (hospital- vs. community-based), typing method(s), total isolates, lineage assignments (with specific flags for L2/Beijing), and MDR counts when reported. Where required, lineage labels were harmonized to standard global lineages (L1–L4, L2/Beijing, etc.). Study quality and reporting transparency were appraised using a simplified JBI-style checklist focusing on sampling clarity, denominator traceability, and typing reproducibility; disagreements were resolved by consensus. The complete list of included studies, with core metadata and key findings, is provided in Supplementary Table S1.

Statistical synthesis focused on pooled proportions with 95% Wilson confidence intervals computed from study-level numerators and denominators aggregated by region and nationally. Chi-square tests (2 × k) were used to quantify inter-regional heterogeneity for Beijing (L2) presence and MDR prevalence. After updating to *n* = 19 and removing double-counting from aggregate “National total” rows, the pooled prevalence of the East-Asian Beijing lineage was 0.58% (18/3085; 95% CI 0.37–0.92%), and the pooled MDR prevalence (excluding MDR-only datasets from denominators) was 0.97% (30/3085; 95% CI 0.68–1.38%). Both outcomes exhibited significant heterogeneity across Mexican regions (χ^2^ (7) = 79.25 for Beijing; χ^2^ (7) = 99.18 for MDR; both *p* < 0.001). The PRISMA 2020 flow counts aligned to these updates are: identified 2318; duplicates removed 898; screened 1420; full text assessed 68; excluded with reasons 49; included 19. The complete PRISMA checklist, database query summary, and the list of included studies (Supplementary Table S1), as well as the statistical outputs (Supplementary Tables S2–S4), are provided in Supplementary Methods.

## 3. Quantitative Synthesis and Statistical Analysis

Based on the 19 eligible studies identified through the PRISMA process, we aggregated 3085 clinical *M. tuberculosis* isolates collected in Mexico between 2010 and 2025. For each outcome, pooled proportions and 95% Wilson intervals were computed from the total numerators/denominators after harmonization. Inter-regional heterogeneity was evaluated with chi-square tests constructed as 2 × k contingency tables of positive vs. negative counts across regions.

Beijing (L2) prevalence. The pooled prevalence of the East-Asian Beijing lineage was 0.58% (18/3085; 95% CI 0.37–0.92%). Beijing-positive isolates were concentrated in Nuevo León and, to a lesser extent, Jalisco, while no Beijing cases were detected in Veracruz, Chiapas, Baja California, Guerrero, San Luis Potosí, or the Multi-state (Central-West) aggregate within the included datasets. The distribution of Beijing across regions was strongly heterogeneous (χ^2^ (7) = 79.25, *p* < 0.001), indicating non-random clustering rather than uniform circulation.

MDR prevalence. Excluding MDR-only datasets from prevalence denominators, the pooled MDR prevalence was 0.97% (30/3085; 95% CI 0.68–1.38%). MDR frequencies varied significantly across regions (χ^2^ (7) = 99.18, *p* < 0.001), with elevated proportions in Nuevo León and Jalisco and very low/zero frequencies in the remaining regions (including Chiapas, Baja California, Guerrero, San Luis Potosí, and Multi-state (Central-West)). Region-wise numerators/denominators and Wilson intervals are provided in Supplementary Tables S2 (Beijing) and S3 (MDR); a summary of national totals and heterogeneity statistics appears in Supplementary Table S4.

Sensitivity and consistency checks. To prevent double-counting, “National total” rows were excluded wherever disaggregated state-level data were available; recalculations after this exclusion produced the estimates above. We verified internal consistency across tables by cross-checking that regional counts sum to national totals used in the pooled calculations and that PRISMA flow counts match the final *k* = 19 included studies.

## 4. Discussion

### 4.1. Statistical Evidence of Regional Heterogeneity

The updated synthesis (*k* = 19) corroborates marked geographic heterogeneity in both lineage distribution and MDR prevalence. Although Beijing (L2) remains rare nationally (0.58%), its concentration in northern Mexico—particularly Nuevo León—suggests localized introductions and restricted transmission chains rather than widespread dissemination. The heterogeneity statistic is large (χ^2^ (7) = 79.25, *p* < 0.001), consistent with non-random clustering. Similarly, national MDR prevalence is low (0.97%) yet non-uniform (χ^2^ (7) = 99.18, *p* < 0.001), with higher signals in Nuevo León and Jalisco and minimal/absent MDR in other regions. Together, these patterns support a landscape of focal micro-epidemics rather than generalized spread.

### 4.2. Implications for Surveillance and Control

The heterogeneity signals argue for regionally tailored surveillance. In northern states, priorities include expanded genomic surveillance (routine MIRU-VNTR plus targeted WGS for clusters and drug-resistant cases), enhanced contact tracing, and integration of clinical/epidemiological metadata to resolve transmission. In regions with absent/minimal Beijing and low MDR, programs can focus on consolidating routine genotyping, strengthening treatment-adherence and comorbidity management (TB–diabetes), and maintaining standardized reporting. Embedding Wilson CIs and formal heterogeneity tests in routine dashboards will improve precision, comparability across jurisdictions, and early detection of changes.

### 4.3. Comparison with Global Patterns and Methodological Considerations

Compared with regions where the Beijing lineage accounts for a substantial fraction of MDR-TB, Mexico shows a distinct profile dominated by Euro-American (L4) sub lineages (T, Haarlem, LAM, X) and only sporadic L2/Beijing detection. In the present synthesis (*k* = 19), Beijing represents 0.58% (18/3085) with concentration in Nuevo León and occasional detection in Jalisco, while no Beijing cases were observed in Veracruz, Chiapas, Baja California, Guerrero, San Luis Potosí, or the Multi-state (Central-West) aggregate. This distribution aligns with historical and demographic forces shaping TB in the Americas and mirrors reports from other Latin-American settings.

Methodologically, included datasets span 2010–2025 and were assembled under heterogeneous, region-specific sampling frames (hospital- vs. community-based recruitment; targeted MDR investigations vs. routine surveillance) and mixed genotyping platforms (spoligotyping, MIRU-VNTR, WGS). Denominators and collection windows were not uniformly reported, introducing spatial and temporal heterogeneity that can influence pooled proportions and between-region contrasts.

Given these constraints, national estimates should be interpreted as an integrative snapshot rather than formal time-trend measures. We did not perform year-stratified meta-analysis or meta-regression because time windows and denominators were inconsistently reported. Nonetheless, applying Wilson 95% CIs to all proportions and chi-square tests across regions yields transparent uncertainty quantification and confirms non-homogeneity in both lineage distribution (χ^2^ (7) = 79.25; *p* < 0.001) and MDR prevalence (χ^2^ (7) = 99.18; *p* < 0.001; pooled 0.97% [30/3085]).

Routine WGS-based surveillance with standardized metadata fields (location, collection period, patient context) would enable random-effects modeling of regional and temporal effects and improve comparability with international datasets. Because time windows and denominators were inconsistently reported, we did not compute year-stratified pooled estimates or meta-regression; we interpret national proportions as an integrative snapshot and recommend prospective, harmonized time-series to quantify genuine temporal shifts [21,22].

### 4.4. Methodological Appraisal of Typing Tools in Mexico

Spoligotyping remains useful for low-cost, large-scale mapping of national lineage structure and for historical comparisons, but its limited discriminatory power constrains outbreak resolution and fine-scale transmission inference. MIRU-VNTR provides greater discrimination for routine surveillance and retrospective cluster analysis; however, performance diminishes in highly clonal contexts and requires standardized laboratory processes across sites. Whole-genome sequencing (WGS) offers the highest resolution to resolve recent transmission, reconstruct phylogenies, and detect resistance-associated variants, while also serving as a quality-control benchmark for lower-resolution methods.

Given the updated national picture—very low Beijing prevalence (0.58%) and low but heterogeneous MDR (0.97%) with hotspots in Nuevo Leon and Jalisco and near-zero signals elsewhere—an operationally efficient tiered approach is warranted: routine MIRU-VNTR for baseline surveillance nationwide, complemented with targeted WGS in (i) cluster investigations, (ii) drug-resistant cases, and (iii) high-incidence or heterogeneity-positive jurisdictions (e.g., Nuevo Leon/Jalisco), scaling bioinformatics capacity as needed [21,22,23,24,25,26]. These choices are consistent with current Mexican practice and datasets [9,13,27].

The reported performance varies by platform and by gene–drug pair. In Mexican datasets using WGS, resistance prediction for first-line drugs generally shows sensitivities ~70–96% and specificities ~90–100%, whereas second-line predictions are more variable and require local calibration against phenotypic testing [9]. These figures align with global practice standards for WGS-based resistance inference [22]. By contrast, spoligotyping and MIRU-VNTR are not resistance tests; their performance pertains to lineage assignment and cluster discrimination rather than sensitivity/specificity for drug resistance. Integrating state/year-resolved numerators and denominators, consistent Wilson CIs, and targeted WGS into routine workflows will maximize epidemiological yield while keeping costs and complexity proportionate to Mexico’s current low-L2/low-MDR landscape.

### 4.5. Limitations

Spatial coverage was uneven, with some states contributing few or no isolates and others dominated by single-center series. Sparse denominators reduce the precision of regional estimates and can bias cluster detection toward better-resourced jurisdictions. Differences in study design (hospital-based versus community surveillance), recruitment strategies, and genotyping platforms further contribute to heterogeneity. These constraints likely attenuate or distort true inter-state contrasts and underscore the need for standardized sampling frames, common metadata fields (location and collection period), and routine WGS-based reporting across Mexican laboratories [27,28,29,30,31].

## 5. Epidemiological Panorama and Risk Factors Associated with Tuberculosis in Mexico

In line with the quantitative synthesis, the following sections contextualize demographic and clinical risk factors that modulate TB transmission and may contribute to the regional heterogeneity observed in lineage distribution and MDR prevalence.

Historically, susceptibility patterns have shifted. In the 1970s, adults over 55 years were the most affected, while infants under one year had the lowest incidence [32]. The broad introduction of Bacillus Calmette–Guérin (BCG) vaccination substantially reduced pediatric cases, reflected in a rate of 3.7 per 100,000 in 2021. Globally and in Mexico, TB incidence in older adults remains disproportionately high, reported to be up to three times greater than in younger populations [33].

Despite sustained efforts in TB control, Mexico remains among the three countries with the highest disease burden in the Americas, along with Brazil and Peru [34]. Between 2000 and 2021, 419,547 cases were reported. In 2019, nearly 23,000 individuals developed TB, with 2600 deaths, corresponding to an incidence rate of 17.7 per 100,000 inhabitants. According to the most recent national surveillance data for Mexico, men account for 63% of notified tuberculosis cases; by age, adults aged 20–64 years represent 76.6% of cases, those ≥65 years 15%, and children 1–14 years 8.4% [35,36]. With the onset of the COVID-19 pandemic, incidence decreased by 4.5% in 2020; however, by 2021 the disease rebounded, with notable increases among adults over 65 years, who reached an incidence of 29.5 per 100,000 inhabitants [37]. Although this apparent increase may suggest a resurgence of tuberculosis transmission, it is more accurately interpreted as a recovery of active TB case detection following the disruption of health services during the COVID-19 pandemic. The temporary decline observed in 2020 likely reflected underdiagnosis and underreporting rather than a true reduction in incidence.

In 2021, the reactivation of surveillance activities, along with the physiological vulnerability of older adults exacerbated by comorbidities, contributed to the higher number of notified cases. This pattern highlights the importance of maintaining uninterrupted TB diagnostic and monitoring programs, even during public health emergencies, to ensure accurate assessment of disease dynamics and timely treatment initiation [38].

**Figure 1:**
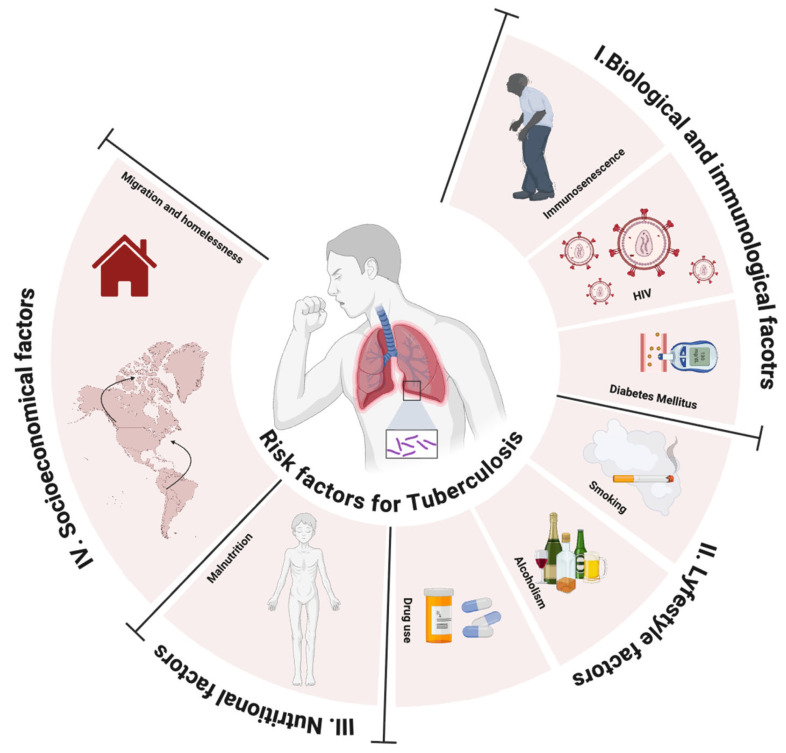
Risk factors for tuberculosis.

In the Mexican context, tuberculosis reflects a complex network of determinants that go beyond infection by Mycobacterium tuberculosis. From this multifactorial interaction, it is possible to establish a classification of the main factors involved, encompassing biological and immunological, lifestyle, nutritional, and socioeconomic aspects that interact with each other and modulate both individual susceptibility and the dynamics of disease transmission (Figure 1) [36].

### 5.1. Biological and Immunological Factors

Immunosenescence. It is an aging-related process that affects both innate and adaptive immunity and contributes to greater susceptibility to infections such as TB [33]. In elderly TB patients, decreased production of interferon-gamma (IFN-γ) has been observed, along with alterations in macrophage phenotype and function, reduced thymic output of T lymphocytes, and diminished capacity to secrete proinflammatory cytokines—all of which compromise the formation of an effective immune response [39].

HIV. HIV infection is well known to cause progressive loss of immune system function, thereby facilitating both the development and reactivation of TB. One of the main immunological effects is the reduction in Th1-type cytokines such as IL-2, IL-12, and IFN-γ, which are essential for macrophage activation and control of *M. tuberculosis*. At the same time, there is an increase in Th2-type cytokines, whose antagonistic activity further undermines macrophage-mediated immunity [40]. As HIV infection progresses, the host’s ability to contain TB diminishes. In moderate stages of HIV disease, CD4+ T-cell lymphocytopenia occurs alongside impaired macrophage differentiation and activation, with the absence of Langhans giant cells. In advanced stages, once acquired immunodeficiency syndrome (AIDS) is established, granuloma formation is severely impaired due to poor cellular recruitment, low CD4+ T-cell counts, and a high bacillary load of acid-fast bacilli [41].

Diabetes Mellitus (DM). Individuals with DM develop granulocytic dysfunction and impaired cellular immunity, characterized by reduced T-cell proliferation and altered delayed-type hypersensitivity [42]. This condition represents a risk factor for the progression of latent TB to active disease and is associated with a higher probability of developing multidrug-resistant TB (MDR-TB), with a significant increase in treatment failure rates [34].

Patients with TB and elevated blood glucose levels have been found to produce higher amounts of IFN-γ compared to non-diabetic individuals. Although this finding may appear paradoxical, it has been proposed that in people with diabetes, the immune response to *M. tuberculosis* may be compromised due to the accumulation of advanced glycation end products (AGEs). These compounds can interfere with T-cell activation and the production of key innate cytokines, such as type 1 cytokines, through mechanisms including delayed proteolysis or the loss of negative feedback regulation [43]. Such immunological alterations increase susceptibility to lower respiratory tract infections. Additionally, impaired innate immunity, combined with the chronic hyperglycemic environment, promotes greater prevalence and severity of infection [44].

This association between TB and diabetes mellitus is particularly relevant in the Mexican context. According to the most recent National Health and Nutrition Survey, the prevalence of diabetes in the country has reached 18.3% [45]. Epidemiological data indicate that between 2000 and 2012, while TB cases not associated with diabetes declined, TB cases linked to this comorbidity showed a sustained increase. These findings highlight the need for differentiated control strategies tailored to this high-risk population [46].

### 5.2. Lifestyle Factors

Smoking. The prevalence of tobacco use in Mexico in 2023 was 15.3%, representing 14.3 million people over the age of 15 worldwide [47]. Smoking increases the likelihood of developing respiratory diseases by inducing ciliary dysfunction and reducing macrophage-mediated immune responses. In addition, it decreases the production of tumor necrosis factor alpha (TNF-α), thereby impairing efficient macrophage activation [34].

TNF-α is a proinflammatory cytokine produced in response to various stimuli, including viral infections. It facilitates the recruitment and activation of mononuclear cells. Along with IFN-γ, epithelial cells, Langhans cells, and macrophages can be organized to form the granuloma [40]. Inadequate granuloma formation is a key step in the progression to active TB. Insufficient production of interleukin-12 (IL-12) and TNF-α limits the activation of natural killer (NK) cells and, consequently, the release of IFN-γ, preventing an effective immune response. As a result, the immune system fails to contain the bacillus, allowing for the clinical manifestation of the disease in the host [34].

The mortality rate among smokers with TB may be up to nine times higher compared to non-smokers; however, it can be reduced by up to 65% upon smoking cessation [48]. Moreover, passive exposure to tobacco smoke poses a significant risk, particularly for children and adolescents, who exhibit greater vulnerability to TB when exposed to the harmful compounds present in second-hand smoke [49].

Alcoholism. Alcohol consumption is closely associated with the induction of oxidative stress and depletion of antioxidant levels within the pulmonary environment. When free radicals are generated, either by a pathogen or as a result of host biological processes, antioxidant defenses are required to counteract their effects. However, chronic alcohol consumption diminishes these protective elements, leading to an inadequate innate immune response that compromises macrophage phagocytosis and microbial clearance. Furthermore, oxidative stress conditions created by alcohol consumption may even favor the growth of *M. tuberculosis* [50].

Alcohol exposure has been shown to impair multiple immune functions of alveolar macrophages, including mobility, adhesion, phagocytic capacity, and superoxide anion production. Alcohol also interferes with the protective role of cytokines and may inhibit their production by monocytes [51]. A particularly relevant alteration in the context of TB is alcohol-induced ciliary dyskinesia, which causes loss of respiratory epithelial cilia and reduces the efficiency of the mucociliary system in clearing pathogens and foreign particles [52].

In addition, prolonged alcohol use compromises the integrity of the alveolar epithelial barrier, increasing its permeability and reducing surfactant production. Pulmonary surfactant plays essential immunological roles by facilitating both phagocytosis and opsonization, processes critical for the containment of *M. tuberculosis* [50].

Drug use. Illicit drug use is closely associated with several TB risk factors, including overcrowding, malnutrition, and HIV coinfection—particularly among people who inject drugs and share needles. The living conditions of this population group increase continuous exposure to high-risk environments. At the immunological level, opioids have been shown to suppress NK cell activity, leading to decreased release of key cytokines such as IFN-γ, TNF-α, and IL-8, which are essential for neutrophil and T-cell recruitment [53]. On the other hand, cocaine use, particularly by inhalation, increases susceptibility to respiratory infections by inhibiting the production of alveolar macrophages and the synthesis of proinflammatory cytokines, including NOS2, affecting the pulmonary antimicrobial response [34].

### 5.3. Nutritional Factors

Malnutrition. Malnutrition represents a major risk factor for the development of TB. It has been estimated that the risk of acquiring the disease under malnourished conditions may increase two- to five-fold in the short term, and this elevated risk can persist for up to a decade after the initial exposure. A reduced body mass index (BMI) has consistently been associated with increased susceptibility to TB [54]. Experimental studies in animal models have demonstrated that protein malnutrition impairs immune responses: mice with insufficient protein intake exhibited reduced production of IFN-γ, TNF-α, and inducible nitric oxide synthases (NOS2), all of which are key molecules in granuloma formation and maintenance. In one study, 100% of mice fed protein-deficient diets died, compared to a 20% mortality rate in those provided with adequate isocaloric diets.

Clinically, malnutrition has also been associated with higher bacillary loads in sputum, increased cavitary lesions, and unfavorable therapeutic outcomes. Severe malnutrition and low BMI during TB treatment have been linked to a four- to five-fold higher risk of mortality compared to eutrophic patients [55].

### 5.4. Socioeconomic Factors

Migration and Homelessness. Homelessness and migration are interrelated conditions that share multiple structural determinants and represent significant risk factors for TB development. Migrants displaced for economic, political, or social reasons often face precarious living conditions, overcrowding, and limited access to healthcare, which heighten vulnerability to TB. The absence of stable housing further exposes these populations to unsafe environments with high pathogen circulation, hindering treatment continuity and favoring community transmission. Both migrants and homeless individuals face overlapping social, economic, and biological risks. In the United States, homelessness has been associated with higher rates of hospital readmission and prolonged hospital stays among TB patients [56]. In Mexico, the border city of Tapachula, Chiapas, exemplifies this dynamic: up to 92% of TB cases reported there occur in migrants. This region also exhibits high HIV prevalence, compounding the epidemiological burden, and hosts a reference hospital for multidrug-resistant TB within its jurisdiction [57].

Consistent with the quantitative synthesis, migration-intense border corridors and urban marginalization coincide with higher lineage diversity and focal MDR prevalence. These conditions facilitate sustained transmission chains, complicate contact tracing, and amplify programmatic delays. Integrating mobility indicators and area-level deprivation into routine molecular surveillance would help anticipate—and interrupt—hotspots of transmission [31,57,58].

## 6. Molecular Methods for Identifying *Mycobacterium tuberculosis* Strains

The CMTB exhibits remarkable genetic diversity, structured in multiple lineages and phylogenetic sublineages. This variability has acquired special relevance in the field of clinical microbiology and public health, due to the association of certain sublineages with specific mutations that confer resistance to first- and second-line anti-TB drugs [5].

Due to the epidemiological and evolutionary characteristics of *M. tuberculosis*, various molecular methods have been developed for the identification, categorization, and phylogenetic analysis of circulating strains, both globally and in specific geographical contexts, such as the case of Mexico. These tools allow for a precise delineation of the population structure of CMTB, facilitate molecular surveillance, and provide evidence on patterns of transmission, evolution, and drug resistance [21].

Currently, there are three molecular methods of special relevance for the epidemiological and phylogenetic study of CMTB, although they are not limited to them: spoligotyping, typing by variable number of tandem repeats of *Mycobacterium* repeats (MIRU-VNTR), and whole-genome sequencing using new generation technologies (WGS/NGS). These tools allow for genetic characterization of strains, establish phylogenetic relationships, identify recent transmission events, and detect mutations associated with antimicrobial resistance with high resolution [13].

Spoligotyping. It is a molecular typing technique used to characterize strains of the MTBC complex, based on the analysis of a specific region of the genome known as CRISPR (Clustered Regularly Interspaced Short Palindromic Repeats), also called direct repeat (DR) region. This region is made up of highly conserved repeating sequences that alternate with single short fragments called spacers. In other bacteria, spacers are usually derived from genetic material from external agents and are part of an adaptive defense system against viruses; however, in *M. tuberculosis*, this region does not fulfill an active immunological function. Even so, the specific combination of spacers present or absent in each strain constitutes a genetic fingerprint that is conserved between generations, which allows this variability to be used as a molecular marker in the spoligotyping technique to identify and phylogenetically classify different strains of the complex [59,60,61].

The classic method consists of amplifying the DR region by polymerase chain reaction (PCR) and then hybridizing the products with specific probes for 43 spacers, generating a binary pattern (present/absent) that allows the lineage and sublineage of the strain to be identified. This pattern is compared with international databases to assign the type of spoligotype and, in many cases, infer the geographic and epidemiological distribution of the strains [59,62,63].

This method has been especially useful in forming databases that would allow the genetic diversity of the CMTB to be seen worldwide. In addition, its potential to be encoded in a numerical form has allowed information to be easily shared. Although spoligotyping was crucial for establishing the first global frameworks of TB molecular epidemiology, its limited discriminatory power prompted the development of more resolutive approaches such as MIRU-VNTR typing [23].

MIRU-VNTR. This methodology is based on the PCR amplification of specific regions of the *M. tuberculosis* genome that contain repetitive tandem sequences, i.e., short fragments of DNA that are repeated one after the other in the same region. These repeats are known as VNTR (Variable Number of Tandem Repeats), and when located in genomic regions called MIRU (Mycobacterial Interspersed Repetitive Units), they allow for highly discriminative molecular typing. The number of repeats at each MIRU locus varies between strains, resulting in a unique numerical profile for each. This variability allows for differentiating and comparing clinical isolates of *M. tuberculosis* with high resolution, being a useful tool in epidemiological, transmission, and outbreak studies [24,25,64,65]. This method is considered to be the current gold standard. The changes present in these sequences evolve slowly, the MIRU-VNTR analysis allows for both epidemiological and phylogenetic study, managing to classify samples at the level of sublineages [25].

While MIRU-VNTR significantly improved the resolution of molecular typing, advances in sequencing technologies have given rise to a new era of genomic epidemiology, where complete genome information allows for unprecedented precision.

WGS-NGS. Genomic methodologies based on the sequencing and computational analysis of an organism’s genetic material have transformed molecular research. Among these, Next-Generation Sequencing (NGS) enables high-throughput analysis of selected genomic regions, whereas Whole-Genome Sequencing (WGS) provides complete genomic coverage, offering an integral perspective of genetic variability and evolutionary dynamics [66].

Since the genome of *M. tuberculosis* comprises approximately 4.4 million base pairs, whole-genomic sequencing (WGS) is a highly suitable tool for TB control. Its application covers both the diagnosis and epidemiological surveillance of strains belonging to lineages of clinical interest, as well as the design of therapeutic strategies [22]. Compared to other molecular typing techniques, WGS offers the greatest resolving power for genotype characterization and analysis of phylogenetic relationships between strains [21].

In addition, it allows for the detection of clinically relevant genomic variants, such as Single-Nucleotide Polymorphisms (SNPs), insertions, and deletions, associated with susceptibility or resistance to drugs. This capacity is essential for the identification of drug-resistant TB cases and for the selection of more effective treatments [9].

When comparing the molecular methodologies used for the characterization of *M. tuberculosis*, it becomes evident that each presents advantages and limitations that determine its applicability in different epidemiological and research contexts [67]. Although spoligotyping has been fundamental for establishing the first global databases and rapidly and economically characterizing lineages, its limited discriminatory power restricts the precise identification of transmission chains or intra-lineage variations. In contrast, MIRU-VNTR offers higher resolution and is considered the reference standard for molecular typing, but its accuracy depends on the number and type of loci analyzed, which leads to discrepancies among laboratories and complicates the harmonization of genetic profiles when equivalent schemes are not used [64]. Conversely, whole-genome sequencing (WGS/NGS) represents the most robust and reproducible methodology, as it allows for the analysis of thousands of single-nucleotide polymorphisms (SNPs), the reconstruction of phylogenetic relationships with high fidelity, and the detection of resistance mutations with near-absolute resolution. However, its implementation requires bioinformatic infrastructure and significantly higher costs, which limit its routine application in resource-constrained settings such as in Mexico [9].

## 7. Prevalent Tuberculosis Genotypes in Mexico: Geographic Distribution and Molecular Characteristics of Predominant Strains

*M. tuberculosis* is a pathogen that has evolved to persist exclusively in human populations [68]. Studies of global strain collections have shown that the human CMTB is composed of separate lineages associated with different regions of the world [69].

On the other hand, phylogenetic research has shown that human-adapted CMTB lineages have a well-defined geographic structure [68]. Migration from Africa likely carried the CMTB with it more than 50,000 years ago, when human populations first crossed the Arabian Peninsula. These ancient migrations were mainly made by land routes and over great distances. This initial diffusion led to the establishment of the CMTB in regions such as the Indian Ocean, Western Europe, northern India, and East Asia [70].

The settlements of human populations in these regions experienced significant growth, creating fertile ground for the expansion and diversification of CMTB strains. With the development of maritime technologies, a turning point in the pathogen’s global journey was also marked. As humans began to explore the world by sea, the CMTB was swept along, reaching new shores and establishing itself in diverse environments. The European era of exploration, characterized by extensive maritime trade and colonization, played a pivotal role in the global spread of the CMTB. European explorers, carriers of the pathogen, introduced it to America, Africa, Asia, and the Middle East. The forced migration of slaves, particularly from Africa to the Americas, further contributed to the spread of TB [69].

The legacy of these historical migrations and population movements is evident in the global distribution of CMTB strains today. While the pathogen originated in Africa, human migrations have shaped its genetic diversity and disease transmission patterns around the world [71].

The population of Mexico is genetically heterogeneous, resulting from admixture between Native American (Amerindian) and European—primarily Spanish—ancestries. Consequently, tuberculosis (TB) in the Americas is predominantly associated with *Mycobacterium tuberculosis* lineage 4 (Euro-American lineage). Nonetheless, the spatial distribution of sublineages within Mexico is heterogeneous [72]. In the central region of Veracruz, sublineage 4.1.1.3 predominates [30], whereas in western states such as Jalisco, sublineage 4.8 is more frequent. These regional patterns likely reflect the influence of historical migration and differences in public health infrastructure [27].

Although the Euro-American lineage is the most widespread, additional lineages have been detected in smaller proportions, including the Indo-Oceanic (L1) and East-Asian (L2) lineages. Their presence suggests multiple introduction events throughout Mexico’s history, likely mediated by trade and migratory exchanges [13]. In Chiapas, the high prevalence of sublineages 4.3.3 and 4.3.4.2—closely related to South American strains—supports the hypothesis of shared evolutionary origins and dissemination through historical transregional routes [72]. Similarly, in states such as Sinaloa, Campeche, Jalisco, Nuevo León, and Yucatán, isolates of L1 and L2 have been identified, reinforcing that the genetic structure of M. tuberculosis in Mexico is shaped by demographic history, migration dynamics, and socioeconomic factors [13].

However, the application and coverage of molecular epidemiology in Mexico remain uneven across regions, which limits a comprehensive understanding of *M. tuberculosis* lineage distribution and transmission dynamics at the national level. Most genomic and molecular typing studies have been concentrated in a few states where research institutions and sequencing infrastructure are available [9]. In contrast, large areas of southern and northern Mexico, including rural and indigenous communities, lack systematic surveillance and molecular characterization programs. This geographic imbalance not only hinders the detection of region-specific lineages and emerging resistant strains but also constrains the development of targeted public health interventions [73].

Mexico has reported more than 20,000 new annual cases of endemic TB, of which approximately 1500 present resistance to at least one of the first-line antibiotics (isoniazid, rifampicin, pyrazinamide, and ethambutol), representing a major epidemiological challenge [74]. The reporting of individual TB cases is the responsibility of each federal entity. The highest incidence rates are predominantly recorded in coastal states such as Baja California, Sinaloa, Veracruz, and Tamaulipas; however, elevated levels are also reported in non-coastal states such as Nuevo León. Although decentralization enables the identification of high-risk areas, the available information on *M. tuberculosis* genotypic variants is inconsistent across regions, limiting precise and comprehensive characterization of the circulating strains in the country [37].

*Mycobacterium tuberculosis* exhibits considerable genetic diversity among its strains, which are classified into lineages and sub-lineages based on genomic features such as the presence or absence of specific oligonucleotide sequences (spoligotypes) or patterns of polymorphic tandem repeats. This classification comprises seven major lineages: Indo-Oceanic, East Asian, East African–Indian, Euro-American, West African/M. africanum I, West African/M. africanum II, and the Horn of Africa. From these, specific sub-lineages arise (Table 2). Their identification is relevant not only for epidemiological surveillance but also for their association with distinct clinical presentations and antimicrobial resistance patterns [5].

**Figure 2:**
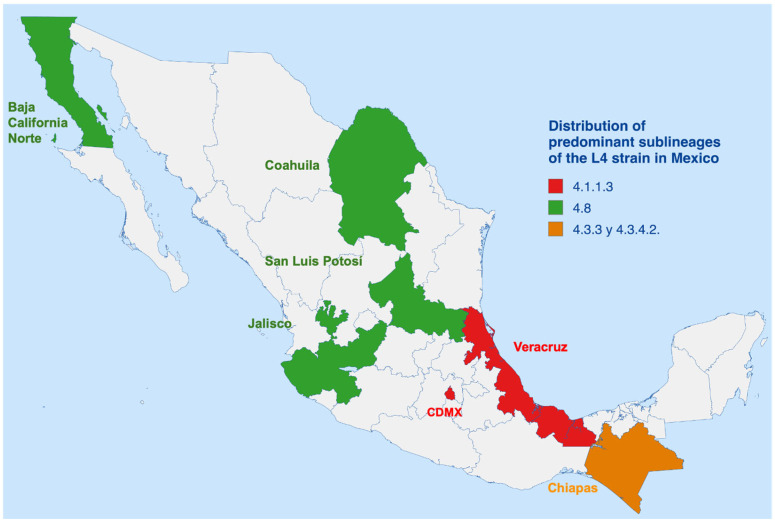
Distribution of predominant sublineages of the L4 strain in Mexico.

In Mexico, circulating strains belong mostly to the Euro-American lineage (L4), although the predominant sublineages vary regionally (Figure 2). In the northern states, such as Baja California and Nuevo León, the most frequent sublineage is T1-SIT53 (4.8), followed by X1-SIT119 (4.1.1.3). In central and southern states such as Jalisco, San Luis Potosí, and Chiapas, T1-SIT53 (4.8) continues to be predominant, although the second most common sublineage differs between states [9,11,18,28,29,75].

In the south-central region, particularly in Mexico City, the X1-SIT119 sublineage (4.1.1.3) predominates, followed by T1-SIT53 (4.8), both of the L4 lineage. However, in areas such as Acapulco, strains belonging to the L1 lineage have been identified, highlighting the presence of EAI2-SIT19 (1.2.1) as the dominant sublineage, followed by EAI3-SIT8 (1.1.2) and T1-SIT5 (4.3.4) [18,76].

Veracruz, one of the states with the highest incidence, shows a high frequency of the X sublineage (4.1.1), followed by Haarlem (4.1.2) and LAM (4.3.3), all belonging to the L4 lineage. In Baja California, on the other hand, the predominance of LAM (4.3), S (4.4),) and Haarlem (4.1.2) hashave been documented, which together represent about 45% of the isolates, without a relevant presence of other lineages [9,77].

The heterogeneous distribution of sublineages within the Euro-American lineage (L4) at the regional level highlights the need for an accurate molecular characterization of this lineage, which is the most prevalent in Mexico. Its predominance has been linked to historical, evolutionary, and epidemiological factors, supported by various scientific studies. In particular, it has been proposed that the introduction of European strains during the colonial period was a key event in their establishment, as demonstrated by genomic analyses that show a close phylogenetic relationship between current Mexican strains and those of Spanish origin [78].

The phylogenetic classification of *M. tuberculosis* is based on specific genetic polymorphisms (Table 3), particularly genes such as *katG* and the gene encoding DNA gyrase subunit A (*gyrA*), which have allowed the strains to be grouped into three main genotypic groups, known as Principal Genotypic Groups (PGG1, PGG2, and PGG3). This categorization provides an evolutionary framework for understanding the genetic diversity of the *Mycobacterium tuberculosis* Complex (CMTB). In this context, the analysis of structural deletion (RD) regions has been key to complement genotypic classification and refine the identification of sublineages [79]. In the case of the Euro-American lineage (L4), most of its sublineages are grouped within PGG2; however, a notable exception has been identified: strains with the RD219 deletion, which belong to PGG3. This difference suggests a more complex evolutionary trajectory within the L4 lineage. In addition to RD219, other relevant genomic deletions in this lineage include RD115, RD174, and RD182, which have been useful in delineating specific sublineages and better understanding pathogen dispersal and adaptation patterns [79,80,81,82].

Beyond their genomic characteristics, strains belonging to the Euro-American lineage also have a distinctive phenotypic profile. Under in vitro conditions, these strains show an accelerated growth rate in liquid media compared to strains from the East Asian (Beijing) and Indo-Oceanic lineages, which could correlate with increased replicative fitness and transmission potential. Functional studies with monocyte-derived human macrophages have shown that these strains induce intermediate levels of proinflammatory cytokines such as TNF-α and IL-12p40, placing them among the immunomodulatory extremes observed in other lineages. In addition, their cell envelope lipid profiles, composed of specific mycolic acids and phosphatidylinositols, contribute to a differential interaction with the receptors of the innate immune system, favoring a moderate inflammatory response and immune evasion mechanisms that can impact the virulence and persistence of the pathogen in the host [83,84].

## 8. Transmission Patterns and Analysis of Vulnerable Populations

TB is a communicable disease that spreads through tiny airborne particles smaller than 5 μm. These particles, generated in the form of aerosols during coughing or sneezing episodes, originate in the airways and laryngeal structures of infected people. Once released, they can remain suspended in the air, especially in poorly ventilated spaces, until they reach the host’s pulmonary alveoli, where the infection process begins [85].

Although coughing is the most recognized form of transmission, it is not the only one. Various studies have shown that other activities such as talking, singing and even breathing can generate aerosols with enough bacillary charge to transmit the disease [86,87,88]. Although coughing emits aerosols from deep regions of the respiratory tree and with a greater infectious load, it occurs on average 26 times per hour, compared to 720 breaths per hour under normal conditions. This indicates that coughing is not a prerequisite for TB infection [89].

To accelerate the reduction in TB incidence and mortality, control strategies must target the main determinants of transmission: the infectious agent, the host and the social factors that favor its spread [90].

TB transmission occurs most often in settings of close and prolonged contact, such as homes and health facilities. However, it can also occur in spaces with casual contact, such as commercial establishments or restaurants. It is estimated that less than 30% of infection cases are directly linked to an identified contact, complicating the traceability of the source of infection, especially in endemic regions [91].

Certain settings are at particularly high risk of transmission, such as hospitals, homeless shelters, prisons, public transport, schools and churches in this kind of places there are voulnerable populations, grups that due to biological, social or enviroment conditions, have an increadsed likelihood of exposure, infection or progession to active disease, as well as poorer clinical outcomes when infected with *M tuberculosis*. Environmental factors such as humidity, poor ventilation and low exposure to ultraviolet radiation contribute to maintaining the viability of bacilli in the environment [92].

Traditionally, it has been assumed that multidrug-resistant TB (MDR-TB) strains have lower transmission capacity compared to susceptible strains. However, a study that analyzed the viability of bacilli present in aerosols generated by cough did not find significant differences between the two clinical forms [93]. Despite this, the spread of MDR-TB may be favored by delays in diagnosis and in the initiation of effective treatment [85].

Beyond these microbiological aspects, individual and population-level risk factors also strongly influence the spread of MDR-TB dissemination. In Mexico, the state of Veracruz reports the highest number of MDR-TB cases nationwide. A regional ranks first in the number of cases. A study conducted in this region revealed that 36% of affected MDR-TB patients also had type 2 diabetes mellitus (T2DM) and 47% had received inadequate prior treatment, emphasizing the need for integrated control strategies that address both therapeutic quality and comorbidity management [9]. These findings underscore the importance of strengthening the predictive and diagnostic use of molecular resistance testing to guide timely, tailored interventions in high-burden settings [73] a comprehensive approach that includes comorbidity control and quality of treatment [9].

In countries with low incidence, transmission is usually sporadic and is concentrated in older adults with reactivation of latent infections. In contrast, in regions with a high TB burden, transmission is active and occurs mainly in young adults and children [94]. Although childhood cases of TB and MDR-TB are rare, their occurrence reflects increased exposure and usually indicates an active chain of transmission. Because children can develop active diseases 6 to 12 months after initial infection, their diagnosis is an epidemiological marker of sustained community transmission [91].

In older people, many cases of TB are due to endogenous reactivation favored by immunosenescence and the presence of comorbidities. However, it has been documented that approximately one-fifth of cases in this population are part of active chains of transmission, evidencing their involvement in the current epidemiology of the disease [33]. Although most older adults with TB reside in their own homes, the incidence of the disease can be increased two- to three-fold in those living in nursing homes [95].

## 9. Role of HIV-TB Co-Infection and Diabetes Mellitus-TB in the Molecular Epidemiology of Tuberculosis

HIV co-infection plays a key role in the molecular epidemiology of TB, modifying both individual susceptibility and transmission dynamics and the evolution of *M. tuberculosis*. Epidemiologically, co-infection with HIV is the main risk factor for progression from latent infection to active disease, with a 15–21 times higher risk in co-infected people compared to those without HIV [96].

This increased risk is due to multiple immunological mechanisms: HIV induces progressive depletion of CD4+ lymphocytes, alters the function of specific T cells against the bacterium, affects macrophage activation and maturation, and compromises antigenic presentation by dendritic cells, which weakens the formation and function of granulomas, facilitating the spread and atypical presentation of TB [97].

In terms of transmission, molecular and epidemiological evidence indicates that TB patients and co-infected with HIV tend to be less infectious than patients without HIV, especially when they have advanced immunosuppression or paucibacillary disease. Contacts of co-infected cases have a lower risk of infection with this microorganism, although heterogeneity is high, and factors such as the presence of pulmonary cavitation and the degree of immunosuppression modify this risk [98]. Population genomics studies have confirmed that HIV co-infection is associated with a reduction in the effective reproductive number of *M. tuberculosis*, i.e., lower transmissibility, regardless of CD4+ count [17].

At the molecular level, HIV co-infection can influence the evolution of mycobacteria. It has been observed that, in immunosuppression contexts, rifampicin-resistant strains with low-fitness mutations can persist and be transmitted, suggesting that selective pressure exerted by the weakened immune system allows fewer fit variants to survive. In addition, there are indications that the immune environment altered by HIV may favor the selection of variants in antigenic regions, which could have implications for vaccine design in populations with high HIV prevalence [99,100].

In Mexico, the lineages of *M. tuberculosis* associated with patients with HIV infection show a high genetic diversity, without there being a single predominant lineage in this population. Molecular studies conducted in HIV-positive patients in Mexico City have identified the presence of multiple lineages, including Haarlem, LAM, T, X, and Beijing, as well as several novel patterns of spoligotyping and MIRU-VNTR, indicating considerable genetic heterogeneity among the strains isolated from these patients. In particular, the T1 lineage has been reported as frequent in immunocompromised patients, although an exclusive association with HIV has not been established [73,101].

Diabetes mellitus (DM) is a well-established risk factor for tuberculosis (TB), with epidemiological data indicating approximately a two- to three-fold increased risk of active TB among individuals with DM, and a modestly increased risk of latent TB infection [102,103]. This association is of relevance in Mexico, a country ranked among those with the highest prevalence of diabetes worldwide and the highest in Latin America, where DM represents a growing public health problem and one of the leading causes of chronic morbidity and mortality [104].

The molecular epidemiology of TB in the context of DM is multifaceted, involving host immune, metabolic, and genetic factors, as well as potential effects on *Mycobacterium tuberculosis* (MTB) lineage distribution and transmission dynamics [105]. From a host perspective, DM alters immune responses to MTB, with evidence of increased neutrophil activation, dysregulated innate immune pathways, and upregulation of gene expression signatures associated with diabetic complications and metabolic dysfunction in TB-DM comorbidity [103,106]. Integrated transcriptomic and metabolomic analyses have identified upregulation of pathways such as IL-17, PI3K-AKT, PPAR, and NOTCH1/JAK/STAT signaling in TB-DM patients, as well as changes in lipid and carbon metabolism, which may contribute to increased susceptibility and adverse outcomes. These molecular changes are consistent across diverse populations, although the core TB gene expression signature remains similar in TB patients with and without DM [106].

Mathematical modeling and population-level analyses suggest that DM can impact TB epidemiology through multiple pathways, including increased progression from latent to active disease, greater infectiousness, and poorer treatment outcomes, with indirect effects on transmission dynamics that may be substantial in regions with high DM prevalence. The population attributable fraction analyses indicate that DM accounts for a significant proportion of TB burden, particularly in low- and moderate-incidence countries [103].

## 10. Application of Whole-Genome Sequencing (WGS) in the Control of Tuberculosis in Mexico

Drug-resistant TB represents one of the main challenges to public health worldwide, and Mexico is no exception. Tackling forms such as MDR-TB and extensively resistant (XDR-TB) require more accurate and advanced diagnostic and surveillance tools. In this scenario, WSG has emerged as a key technology to improve the detection of mutations associated with resistance, optimize therapeutic regimens, and strengthen epidemiological surveillance of *M. tuberculosis* strains with complex genotypic profiles [107].

Unlike conventional methods such as phenotypic and other genotypic methods (such as Sanger sequencing), WGS provides higher resolution to identify complex mutations and effectively trace chains of transmission. This is crucial for adjusting treatments, especially in cases of MDR-TB and XDR-TB, where standard treatment often fails [9]. WGS can also identify bacterial lineages and establish epidemiological connections that help design more effective control strategies [26].

In Mexico, the WGS is still in the early stages of implementation, but studies have shown its usefulness. For example, research in regions with a high incidence of resistant TB has shown that WGS is able to detect mutations that are not easily identified by traditional methods [108]. This is particularly important in a country with high genetic diversity of *M. tuberculosis*, whose regional variability limits the efficacy of standardized diagnostic techniques and highlights the need for more advanced technologies such as WGS [13].

Likewise, the use of the WGS in Mexico has allowed us to obtain a better understanding of the dynamics of TB transmission, identifying clusters of cases that share a common source. This information is critical to guide more effective health interventions in vulnerable and high-transmission communities [14].

One of the most representative studies in this field was carried out by Madrazo-Moya [9], who analyzed 81 clinical isolates of *M. tuberculosis* in an endemic region of the country. The study identified four main lineages (L1, L2, L3, and L4), the latter being the predominant one, with a frequency of 90% of the isolates. Likewise, six transmission clusters were identified, highlighting the TC6 cluster, made up of 13 isolates with resistance to multiple drugs. The analysis using WGS allowed for the detection of mutations associated with resistance to first- and second-line drugs. In that study, the sensitivity to predict resistance to isoniazid, rifampicin, ethambutol, pyrazinamide, and streptomycin ranged from 71% to 96%, while the specificity ranged from 90% to 100%. Variants related to second-line drug resistance were also identified, which allowed some cases to be classified as XDR-TB [9].

In addition, Zenteno-Cuevas [27] carried out a phylogenetic analysis of clinical isolates from the state of Veracruz, using WGS to identify mutations associated with drug resistance. This study confirmed the usefulness of the WGS as an advanced molecular tool for the diagnosis of resistant TB in the country [14].

Despite its benefits, widespread adoption of WGS in Mexico faces significant barriers, including the high cost of equipment, limited public health infrastructure, and a shortage of trained bioinformatics personnel. However, the progressive reduction in technological costs and the development of more accessible computational platforms are facilitating their incorporation. It is expected that, with increased investments in infrastructure and specialized training, the WGS will become a tool for routine use in public health laboratories, significantly strengthening TB control in Mexico [21].

In addition to the progressive implementation of whole-genome sequencing (WGS), other molecular methodologies continue to play a fundamental role in the control of tuberculosis in Mexico. Among these are conventional and real-time polymerase chain reaction (PCR), used for the direct detection of the *M. tuberculosis* complex and mutations associated with drug resistance and isothermal amplification techniques, which represent promising alternatives for resource-limited settings [67,109].

## 11. Factors Involved in the Molecular Diversity of Tuberculosis

CMTB is made up of a group of bacteria that present different ranges of pathogenicity and different degrees of severity. They can grow slowly or quickly, depending on their molecular difference. Geographic factors are of great importance in this molecular differentiation, as the burden of disease varies. An example of this is the affinity for Southeast Asia [110].

Although the strains of CMTB have molecular differences, they all derive from a common ancestor, which shows their close phylogenetic relationship. The variations observed between strains usually correspond to SNPs, most of which do not involve changes in the amino acid sequence. These SNPs accumulate slowly yet remain biologically significant, as certain variants are linked to strain transmissibility and the emergence of drug resistance. In 1997, a survey of >600 *Mycobacterium tuberculosis* isolates enabled classification into major lineages: groups 1 and 2 constitute the predominant clades, whereas group 3 represents a more recently evolved lineage [67].

On the other hand, Long-Chain Polymorphisms (LSPs) allow for the phylogenetic classification of *M. tuberculosis* into different lineages. These structural polymorphisms involve specific regions of the chromosome, with the RD (*Region of Difference*) deletion sequences being fundamental for the study and differentiation of strains. Through the analysis of these regions, it is possible to establish phylogenetic relationships and determine specific lineages. For example, although there is a high genomic homology among the CMTB strains, some are exclusive to certain species and hosts: *M. tuberculosis*, *M. africanum*, and *M. canettii* are human pathogens; *M. microti* mainly infects rodents; *M. pinnipedii* to seals; *M. caprae* to goats; and *M. bovis* has a wider range of hosts, including farm animals and humans. In addition to the classifications mentioned above, there is a classification by lineages, which are subdivided into 6: Indo-Oceanic, East Asian, East Africa-India, and Euro-American, in addition to 2 classically identified lineages for *Mycobacterium africanum*: West Africa I and West Africa II [67].

Using the WGS, the total variation in the genome, focused on the chain of transmission, is analyzed. Through this technique, up to 9 lineages have been identified, each of which has different behaviors: lineages 1, 2, 3, 4, and 7 are mainly associated with pathologies, while lineages 5 and 6 are less virulent. Although it is a broader classification, they have a better understanding, since they start from the original strain, according to various theories, almost more than 70,000 years old [110].

The most prevalent lineage globally is lineage 2, with the Beijing strain being the most common. It is a strain with an extremely interesting behavior, since it is variable based on the population or area where it is found, which makes it very diverse in its mechanism and resistance to drugs [111].

## 12. Public Health Policies and Their Relationship with the Molecular Epidemiology of Tuberculosis

National and international strategies have been instrumental in TB containment and monitoring. The World Health Organization (WHO) set a goal of ending the disease by 2035, to reduce incidence by 90% (less than 10 cases per 100,000 inhabitants) and reduce mortality by 95%, taking 2015 levels as a reference. To achieve these goals, measures such as vaccination and preventive treatment in vulnerable populations are promoted [112].

In Mexico, molecular epidemiology directly supports programmatic decision-making by enabling early cluster detection, inference of recent transmission, and rapid resistance prediction to tailor regimens. Spoligotyping is inexpensive and scalable for baseline lineage mapping, MIRU-VNTR enhances routine surveillance and retrospective cluster analysis, and WGS resolves transmission links and resistance profiles with the highest accuracy. A pragmatic, tiered deployment—baseline spoligotyping/MIRU-VNTR with targeted WGS for clusters, MDR-TB, and high-incidence locales—aligns with current resources while strengthening the evidence base for targeted interventions [21,23,24,25], with concrete applications already reported in Mexico [9,13,27].

Currently, national coverage reaches around 95% in children under five years of age. The application is mandatory for in all newborns in Mexico, to prevent severe forms of the disease: BCG confers between 65% and 85% protection against meningeal and miliary forms, and about 50% against the pulmonary form [113].

According to NOM-036-SSA2-2012, BCG must be applied to all newborns or in the first contact with health services before the age of one. It can also be administered until before the age of 4, and even until the age of 14, if it is proven that it has not been previously received. In addition to vaccination, there are other prevention and control measures. Isoniazid is used as a preventive therapy in people in contact with patients with active TB, provided that the disease has been ruled out. It is also indicated in immuno-compromised patients who meet the established criteria [114].

Among the main risk factors are diabetes mellitus and HIV. The Official Mexican Standard for the Prevention and Control of TB (NOM-006-SSA2-2013) establishes that respiratory symptoms in patients with diabetes must be intentionally investigated in each medical consultation. On the other hand, in any case diagnosed with TB, HIV testing should be routinely offered, because this co-infection increases the probability of pulmonary and extrapulmonary forms. The epidemiological surveillance system includes mandatory notification to the Ministry of Health. Cases of meningeal TB must be reported within 24 h, while other forms of TB are reported weekly or monthly. Confirmed or suspected cases of multidrug-resistant TB (MDR-TB) should also be reported [115].

Confirmation of MDR-TB requires demonstrating concurrent resistance to isoniazid and rifampicin by drug-resistance testing. There is a national inclusion in the guide for the management of these cases that includes diagnosis, registration, treatment, contact tracing and follow-up [74].

Standard biosecurity measures are applied at all levels of IMSS care: hand washing, use of gloves, mask, glasses, waterproof gown, and collectors, according to the procedure carried out. Since TB is transmitted by air, additional precautions are in place, such as keeping the patient in respiratory isolation in a ventilated room, limiting their transfers, and recommending the use of N95 masks [116].

The Mexican health system has relevant strengths for TB control, including universal access to diagnosis and treatment, the availability of national guidelines that standardize procedures, and a robust surveillance system. In this context, the relationship between public health policies and the molecular epidemiology of TB in Mexico is close and bidirectional. The application of molecular tools provides critical information for decision-making by allowing for the identification of transmission patterns, predominant lineages, foci of resistance, and geographical groupings, which facilitates targeting interventions and optimizes the use of available resources [117].

Several studies in Mexico have shown that molecular tools such as MIRU-VNTR, spoligotyping, and genomic sequencing allow for the characterization of the genetic diversity of *M. tuberculosis*, the identification of lineages associated with primary resistance and multidrug resistance, and the mapping of the spatial distribution of cases. For example, the high prevalence of lineages such as Haarlem and LAM, and their association with the transmission of primary resistance and MDR-TB, represent specific challenges for control programs, which require strategies adapted to the local molecular and epidemiological reality [13,31,111].

The identification of transmission networks and molecular clusters, particularly in regions with a high prevalence of comorbidities such as diabetes mellitus, has allowed public health programs to prioritize actions in areas of higher risk and adjust surveillance and treatment strategies, as documented in Veracruz and Nuevo León [58,118]. Likewise, spatial and molecular analysis has shown the influence of social, migratory, and economic factors on the spread of TB, underlining the need for intersectoral and regionally adapted policies [94,119].

Although the implementation of molecular surveillance in Mexico still faces technological and coverage limitations, it has been recognized as essential for the detection of outbreaks, the identification of transmission chains, and the response to resistant strains. The expansion of genomic databases and the integration of epidemiological and geospatial information will strengthen the capacity of national programs to contain TB and adapt public health policies to emerging challenges [13,31].

Emerging biotechnological approaches, such as the use of mycobacteriophages and phage-derived enzymes, are gaining attention as potential complementary strategies for treating multidrug-resistant *M. tuberculosis* strains. Lytic phages such as DS6A, TM4, D29, and vB_MapS_FF47 have been engineered to deliver reporter genes or shuttle plasmids that can both diagnose and kill bacilli, and DS6A has demonstrated the ability to infect and lyse MTB complex strains in vitro and in animal models. In guinea pig studies, sub-cutaneous administration of DS6A, GR-21/T, and My-327 significantly reduced lung and spleen bacillary loads, with DS6A showing the greatest therapeutic impact. Temperate phages like ZoeJ, BPs, and Muddy have been adapted into lytic derivatives and combined in phage cocktails to broaden host range and prevent resistance; clinical reports describe successful intravenous phage therapy in cystic fibrosis patients infected with *M. abscessus*, suggesting that a similar strategy could be applied to TB [119].

Phage-derived endolysins (Lysin ALysin A and Lysin BLysin B) further enhance bactericidal activity by degrading the mycobacterial cell wall, and recombinant Lysin BLysin B from phage D29 has shown antimicrobial effects against *M. ulcerans* and other mycobacteria. Synergistic approaches that pair phages or lysins with first-line antibiotics (e.g., isoniazid, rifampicin) increase drug penetration and reduce bacterial viability, offering a potential route to shorten therapy duration and overcome dormancy-related drug tolerance. Together, these strategies illustrate a multifaceted therapeutic arsenal that could complement, and in some cases replace, traditional TB treatment regimens [120].

## Figures and Tables

**Table 1:** Genes and mutations associated with drug resistance in *Mycobacterium tuberculosis* in Mexico.

Drug	Genes Involved	Mutations Reported in Mexico
Isoniazid (INH)	*katG*, *inhA*, *fabG1*, *oxyR-ahpC*	*katG*315S/T; mutation on the promoter *inhA* y *oxyR-ahpC*
Rifampicin (RIF)	*rpoB*	RRDR mutations: codons 531 (S531L), 526, 516
Pyrazinamide (PZA)	*pncA*, *rpsA*	Heterogeneous mutations in *pncA*
Ethambutol (EMB)	*embB*	Mutation in codon 306
Streptomycin (SM)	*rpsL*, *rrs*, *gidB*	*rpsL* (K43R, K88R); mutations in *rrs*; polymorphisms in *gidB*

**Table 2:** Lineages and sub-lineages of the *Mycobacterium tuberculosis* complex.

Lineage	Identification Characteristics	Sub-Linage	
Indo-Oceanic L1	RD239	1.1	1.1.1 1.1.2 1.1.3
		1.2	1.2.1 1.2.2
East Asia L2	RD105 (Beijing) RD142 (2.2.1.2) RD150 (2.2.1.1) RD181 RD207	2.1	
		2.2	2.2.1 2.2.1.1 2.2.2.2
East Africa-India L3	RD750 (CAS)	3.1	3.1.1 3.1.2.1 3.1.2.2
Euro-American L4	RD182 (4.1.2.1) RD 183 (4.1.1.1) RD193 (4.1.1.3) RD115 RD174 RD761 (4.3.2.1) RD726 (4.6.2) RD724 (4.6.1) RD219 (4.8)	4.1	4.1.1.1 (X-Type) 4.1.1.2 (X-Type) 4.1.1.3 (X-Type) 4.1.2 4.1.2.1 (Haarlem)
		4.2	4.2.1 (Ural) 4.2.2.1 (TUR)
		4.3 (LAM)	4.3.1 4.3.2 4.3.2.1 4.3.3 4.3.4.1 4.3.4.2 4.3.4.2.1
		4.4 (S-Type)	
		4.5	
		4.6	
		4.6.1	4.6.1 4.6.2 (Cameroon)
		4.7	
		4.8	
		4.9	
West Africa *(M. Africanum I)* L5	RD711	5.1	
		5.2	
		5.3	
West Africa *(M. Africanum II)* L6	BOVIS RD702	6.1	
		6.2	
		6.3	
L7	RD702	---	

**Table 3:** Geographic distribution of *Mycobacterium tuberculosis* resistance profiles in Mexico.

Strain	Sub Lineage	Resistance Gene	States of Origin
4.1.1.3	(X-type)	INH, RIF, STR, PZA, EMB	Veracruz, Estado de México, Puebla, Guerrero, Tabasco.
4.1.2.1	(Haarlem)	INH, RIF	Nuevo León, Baja California, Jalisco
4.3.3	(LAM)	INH, STR	Baja California, Sinaloa
4.4	(S)	PZA	Veracruz, Chiapas
4.3	(Ancestral LAM)	EMB	Chihuahua, Michoacán

## Data Availability

All data extracted from the included studies and used for the pooled analyses are provided and summarized in Supplementary Tables S1–S4.

## Supplementary Materials

The following supporting information can be downloaded at https://www.mdpi.com/article/10.3390/microorganisms13112453/s1. Table S1. Studies Included in the Systematic Review and Meta-Analysis (n = 19). Table S2. Regional Frequency of Beijing Lineage in Mexico (2010–2025). Table S3. Regional Prevalence of Multidrug-Resistant (MDR) *M. tuberculosis* (excluding MDR-only datasets). Table S4. Statistical Synthesis of Beijing and MDR Variability Across Mexican Regions.

## Abbreviations

The following abbreviations are used in this manuscript:

TB	Tuberculosis
BCG	Bacillus Calmette–Guérin
MDR-TB	Multidrug-resistant tuberculosis
XDR-TB	Extensively drug-resistant tuberculosis
INH	Isoniazid
RIF	Rifampicin
PZA	Pyrazinamide
EMB	Ethambutol
SM	Streptomycin
MIRU-VNTR	Mycobacterial Interspersed Repetitive Unit—Variable Number Tandem Repeat
WGS	Whole-Genome Sequencing
NGS	Next-Generation Sequencing
SNP	Single-Nucleotide Polymorphism
RD	Region of Difference
PGG	Principal Genotypic Group
HIV	Human Immunodeficiency Virus
IFN-γ	Interferon gamma
TNF-α	Tumor Necrosis Factor alfa
IL	Interleukin
NK	Natural Killer
AGEs	Advanced Glycation End products
NOS_2_	Nitric Oxide Synthase 2
IMSS	Instituto Mexicano del Seguro Social
NOM	Norma Oficial Mexicana
DOF	Diario Oficial de la Federación
CMTB	Complex *Mycobacterium tuberculosis*
L1–L7	Lineages of *Mycobacterium tuberculosis*
SIT	Spoligotype International Type
