# Supplementary material for: Molecular Epidemiology of Mycobacterium tuberculosis in Mexico
Source: Microorganisms. 2025 Oct 25;13(11):2453. doi: 10.3390/microorganisms13112453 (PMC12654501; doi:10.3390/microorganisms13112453)
Supplement: Supplementary file 1 [file microorganisms-13-02453-s001.zip › microorganisms-3880103-supplementary.pdf]

Supplementary Material

Systematic Review and PRISMA 2020 Framework

Information sources and dates

This systematic review was designed and reported in accordance with PRISMA 2020. Searches covered January 2010–April 2025 in PubMed, Scopus, Google Scholar, and the MDPI Open Access Database. No language filters were applied at the search stage; only peer-reviewed, indexed publications were eligible. Reference lists of included papers were hand-screened to identify additional records.

Search strategy

The primary Boolean string was: *(Mycobacterium tuberculosis OR MTB) AND (Mexico OR Mexican) AND (spoligotype OR MIRU-VNTR OR "whole genome sequencing" OR WGS OR lineage OR genotype OR sublineage OR drug resistance)*.

Selection process (updated PRISMA counts)

The search identified 2,318 records worldwide. After automated and manual deduplication, 1,420 unique records remained for title/abstract screening. We evaluated 68 full-text articles on Mexican clinical isolates; 19 studies met all inclusion criteria and were incorporated into the final synthesis (k = 19). To prevent double counting in pooled estimates, aggregate "National total" rows were not used when state-level data were available for the same studies/periods.

PRISMA 2020 flow (updated):
Identified (total) = 2,318; Duplicates removed = 898; After deduplication (screened) = 1,420; Excluded at title/abstract = 1,352; Full-text assessed = 68; Full-text excluded (with reasons) = 49; Included in synthesis = 19.

Eligibility criteria

Inclusion. (i) Clinical isolates of *M. tuberculosis* obtained in Mexico; (ii) molecular typing by spoligotyping, MIRU-VNTR, or WGS; (iii) quantitative reporting of lineage distribution and/or drug-resistance metrics with explicit numerators/denominators.
Exclusion. Datasets with <10 isolates; reviews/commentaries without primary quantitative data; MDR-only datasets were excluded from pooled MDR prevalence denominators (but could be used descriptively for mutation summaries where applicable).

Data extraction and harmonization

For each study we extracted: study period, state/region, sampling frame (hospital vs community), typing method(s), total isolates (n), lineage assignments (with explicit L2/Beijing flag), and MDR counts when reported. Lineage labels were harmonized to global lineages (L1–L4; L2/Beijing). Deduplication relied on DOI when available; otherwise, a normalized title+year+first_author key was used. Where state-level denominators were available alongside an aggregated National total, the latter was omitted from pooled numerators/denominators to avoid double counting.

Supplementary Table S1. Studies Included in the Systematic Review and Meta-Analysis (n = 19)

No.	Reference (full title)	Year	Region/State	n isolates	Method	Key lineages or findings	MDR (%)
1	Molina-Torres C.A. et al., "Mycobacterium tuberculosis spoligotypes in Monterrey, Mexico," *International Journal of Tuberculosis and Lung Disease*	2010	Nuevo León (Monterrey)	180	Spoligotyping	SIT53 (23.8%), SIT119 (15.5%) predominant; Euro-American lineage dominant	0
2	Flores-López C.A. et al., "Molecular epidemiology of Mycobacterium tuberculosis in Baja California, Mexico: A result of human migration?" *Infection, Genetics and Evolution*	2017	Baja California	140	MIRU-VNTR	Haarlem/T1 predominant; no Beijing isolates detected	0
3	Rendón-Bautista A. et al., "Characterization of genetic diversity and clonal complexes by whole genome sequencing of Mycobacterium tuberculosis isolates from Jalisco, Mexico," *Frontiers in Microbiology*	2021	Jalisco	32	Whole Genome Sequencing (WGS)	L4.1.2 and L4.8 predominant; Beijing 3%	6.3
4	Flores-Treviño S. et al., "Genetic diversity of Mycobacterium tuberculosis in Guadalajara, Jalisco, Mexico," *BMC Infectious Diseases*	2015	Jalisco (Guadalajara)	68	Spoligotyping + RFLP	T (38%), Haarlem (18%), LAM (18%), Beijing (1.5%)	1.5
5	Rojas J. et al., "Genetic diversity of drug and multidrugresistant Mycobacterium tuberculosis circulating in Veracruz, Mexico," *Infection, Genetics and Evolution*	2018	Veracruz	112	Spoligotyping + MIRU-VNTR	T1 (24%), LAM (16%), Haarlem (15%), SIT53 dominant	3.6
6	Blanco-Guillot F. et al., "Genotyping and spatial analysis of pulmonary tuberculosis and diabetes cases in the state of Veracruz, Mexico, Mexico," *PLoS ONE*	2018	Veracruz (Orizaba)	1105	GIS + Spoligotyping	Clustering of T1/LAM lineages; TB-DM spatial association	—
7	Esdras M. et al., "Whole genome sequencing analysis of Mycobacterium tuberculosis isolates from Veracruz, Mexico," *Brazilian Journal of Infectious Diseases*	2022	Veracruz (Central)	25	Whole Genome Sequencing (WGS)	L4.1.2 and LAM sublineages; low MDR frequency	4.0
8	Molina-Torres A. "Genetic Diversity of Mycobacterium tuberculosis Isolates From an Amerindian Population in Chiapas, México," *Frontiers in Cellular and Infection Microbiology*	2022	Chiapas	73	Spoligotyping + WGS	SIT53 (19%), SIT42 (14%), L4.8/L4.3.3 (LAM)	0
9	Nava-Aguilera E. et al., "Clustering of Mycobacterium tuberculosis Cases in Acapulco: Spoligotyping and Risk Factors," *Clinical and Developmental Immunology*	2011	Guerrero (Acapulco)	273	Spoligotyping	Clustering of T and LAM types; urban transmission	2.2
10	Rendón-Martínez L.M. et al., "Spoligotyping and geospatial analysis of	2025	Nuevo León	151	Spoligotyping + GIS	T1 (35%), X1 (19%), LAM	9.9

	Mycobacterium tuberculosis in northern Mexico," *Scientific Reports*					(10%), Beijing (3.3%)	
11	Cabrera-Vera L. et al., "Comparative Mycobacterium tuberculosis Spoligotype Distribution in Mexico" *Journal of Clinical Microbiology*	2013	Nuevo León and Mexico City	414	Spoligotyping	SIT53 prevalent in north; SIT119 dominant in center	1.2
12	Lope-Rocha E. et al., "Genetic diversity of the Mycobacterium tuberculosis Complex in San Luis Potosí, México," *BMC Research Notes*	2013	San Luis Potosí	237	Spoligotyping	LAM (34%), T (23%), X (12%)	0
13	Torres-Rojas D. et al., "Molecular genotyping of Mycobacterium tuberculosis isolates from central and southern Mexico," *Revista Latinoamericana de Microbiología*	2010	Central-South	95	Spoligotyping	Euro-American lineage dominant	—
14	Negrete Paz A. et al., "Whole-Genome Sequence-Based Diversity of Mycobacterium tuberculosis Strains Isolated from a CentralWestern Region of Mexico" *Pathogens*	2025	Michoacán	77	Whole Genome Sequencing (WGS)	L4.1.1.3 (X) and L4.1.2.1 (LAM); MDR 16%	16.3
15	Hernández-González M. et al., "Whole-genome sequencing for the rapid detection of multidrug-resistant tuberculosis in Mexico," *PLoS ONE*	2019	Multi-site (Mexico)	81	Whole Genome Sequencing (WGS)	L4 ≈90%, validation of MDR prediction algorithm	3.7
16	Zenteno- Cuevas R. et al., "rpoB, katG and inhA mutations in multi-drug resistant strains of Mycobacterium tuberculosis clinical isolates from southeast Mexico," *Enfermedades Infecciosas y Microbiología Clínica*	2019	South Mexico	74	Mutation analysis	rpoB531L and katG315T frequent; MDR dataset	100 (MDR only)
17	Zacarias- Hernandez. et al., "Geographical location and genotyping analysis of pulmonary tuberculosis in the state of Nuevo Leon, Mexico," *Scientific Reports*	2025	Nuevo León	151	Spoligotyping + GIS	Beijing 3.3%; MDR 9.9%	9.9
18	Flores- Arechiga A. et al ., "Molecular epidemiology and drug resistance of Mycobacterium tuberculosis in a tertiary care hospital in northeastern Mexico *Journal of Infection in Developing Countries*	2023	Nuevo León	190	WGS	L4.1.2 dominant; Beijing rare	5.3
19	Hernández-Pérez R. et al., "Genomic epidemiology analysis of drug-resistant Mycobacterium tuberculosis distributed in Mexico," *PLoS ONE*	2023	National (17 states)	85	Whole Genome Sequencing (WGS)	L4 96%, L2 3%; DR 40%, MDR 5%	5.0

Supplementary Table S1 – Summary Description

The 19 studies included in this review span 2010–2025 and comprise both single-state and multi-state cohorts. The largest state-specific sample sizes derive from Veracruz (Orizaba) (~1,105 isolates) and Nuevo León (~492), with additional sizable series from Guerrero (Acapulco) (273) and San Luis Potosí (237). One multi-site dataset integrates isolates from Nuevo León and Mexico City (~414), providing broader geographic coverage. Across studies, Euro-American (L4) sublineages (e.g., T, Haarlem, LAM, X) predominate nationwide, while Beijing (L2) appears sporadically, concentrated in northern Mexico (notably Nuevo León), consistent with the pooled national estimate of 0.58% (18/3,085).

Methodologically, spoligotyping features in 11/19 studies (sometimes coupled with GIS or legacy RFLP), WGS in 7/19, and MIRU-VNTR in 2/19, with several works combining

platforms (e.g., spoligotyping + WGS, MIRU-VNTR + spoligotyping). One dataset is MDR-only (excluded from pooled MDR denominators but informative for mutation patterns), wherein classic resistance-associated variants such as rpoB S531L and katG S315T are prominent. Overall, the S1 corpus supports a national picture of low L2/Beijing prevalence and low but heterogeneous MDR, setting up the regional analyses and pooled statistics reported in Supplementary Tables S2–S4.

Supplementary Table S2. Regional Frequency of Beijing Lineage in Mexico (2010–2025)

Region	Beijing-positive (x)	Total isolates (n)	Proportion (%)	95% CI (Wilson)
Nuevo León	17	521	3.26	2.05–5.16
Jalisco	1	100	1.00	0.18–5.45
Veracruz	0	1,242	0.00	0.00–0.31
Chiapas	0	73	0.00	0.00–5.00
Baja California	0	140	0.00	0.00–2.67
Guerrero	0	273	0.00	0.00–1.39
San Luis Potosí	0	237	0.00	0.00–1.60
Multi-state (Central-West)	0	499	0.00	0.00–0.76
**Total (pooled)**	**18**	**3,085**	**0.58**	**0.37–0.92**

Supplementary Table S3. Regional Prevalence of Multidrug-Resistant (MDR) *M. tuberculosis* (excluding MDR-only datasets)

Region	MDR-positive (x)	Total isolates (n)	Proportion (%)	95% CI (Wilson)
Nuevo León	25	521	4.80	3.27–6.99
Jalisco	2	100	2.00	0.55–7.00
Veracruz	3	1,242	0.24	0.08–0.71
Chiapas	0	73	0.00	0.00–4.97
Baja California	0	140	0.00	0.00–2.67
Guerrero	0	273	0.00	0.00–1.39
San Luis Potosí	0	237	0.00	0.00–1.60
Multi-state (Central-West)	0	499	0.00	0.00–0.76
**Total (pooled)**	**30**	**3,085**	**0.97**	**0.68–1.38**

Supplementary Table S4. Statistical Synthesis of Beijing and MDR Variability Across Mexican Regions

Outcome	x	n	Pooled proportion (%)	95% CI (Wilson)	Chi-square	df
Beijing (L2)	18	3,085	0.58	0.37–0.92	79.25	7
MDR (excl. MDR-only)	30	3,085	0.97	0.68–1.38	99.18	7

Supplementary Tables S2–S4 – Statistical Analysis and Interpretation

Table S2 (Beijing, L2). Using pooled numerators/denominators and Wilson 95% CIs, the national prevalence of Beijing was 0.58% (18/3,085; 95% CI 0.37–0.92%). Positives were concentrated in Nuevo León (17/521; 3.26%; 95% CI 2.05–5.16%) and detected sporadically in Jalisco (1/100; 1.00%; 95% CI 0.18–5.45%). No Beijing isolates were observed in Veracruz (0/1,242; 95% CI 0.00–0.31%), Chiapas (0/73; 0.00–5.00%), Baja California (0/140; 0.00–2.67%), Guerrero (0/273; 0.00–1.39%), San Luis Potosí (0/237; 0.00–1.60%), or the Multi-state (Central-West) aggregate (0/499; 0.00–0.76%). A 2×k chi-square test confirmed strong inter-regional heterogeneity ($\chi^2(7)=79.25$, $p<0.001$), driven largely by the concentration in Nuevo León. “National total” aggregates were excluded from pooling to avoid double counting.

Table S3 (MDR prevalence). Excluding MDR-only datasets from denominators, the national MDR prevalence was 0.97% (30/3,085; 95% CI 0.68–1.38%). MDR was highest in Nuevo León (25/521; 4.80%; 95% CI 3.27–6.99%) and present in Jalisco (2/100; 2.00%; 95% CI 0.55–7.00%) and Veracruz (3/1,242; 0.24%; 95% CI 0.08–0.71%), with zero events in Chiapas (0/73; 0.00–4.97%), Baja California (0/140; 0.00–2.67%), Guerrero (0/273; 0.00–1.39%), San Luis Potosí (0/237; 0.00–1.60%), and Multi-state (Central-West) (0/499; 0.00–0.76%). The 2×k chi-square indicated marked heterogeneity ($\chi^2(7)=99.18$, $p<0.001$), reflecting focal elevation in Nuevo León (and to a lesser extent Jalisco) against uniformly low levels elsewhere. “National total” aggregates were excluded from pooling.

Table S4 (national summary and heterogeneity). Collating S2–S3, Beijing (L2) is rare nationally (0.58%) yet non-uniformly distributed ($\chi^2(7)=79.25$, $p<0.001$), and MDR is low overall (0.97%) but likewise heterogeneous ($\chi^2(7)=99.18$, $p<0.001$). Wilson intervals provide comparable uncertainty bounds across regions with disparate denominators, and exclusion of MDR-only datasets from denominators prevents inflation of MDR prevalence. These findings indicate localized introductions/clusters (notably Nuevo León, sporadically Jalisco) within a broader landscape dominated by Euro-American (L4) lineages and low MDR prevalence.
